# Intravenous Flat-Detector Computed Tomography Angiography for Symptomatic Cerebral Vasospasm following Aneurysmal Subarachnoid Hemorrhage

**DOI:** 10.1155/2014/315960

**Published:** 2014-10-14

**Authors:** Jin Pyeong Jeon, Seung Hun Sheen, Yong-Jun Cho

**Affiliations:** ^1^Department of Neurosurgery, Seoul National University College of Medicine, Seoul, Republic of Korea; ^2^Department of Neurosurgery, Bundang Jesaeng Hospital, Kwandong University College of Medicine, Bundang, Republic of Korea; ^3^Department of Neurosurgery, Hallym University College of Medicine, Chuncheon, Republic of Korea

## Abstract

The study evaluated the diagnostic accuracy of intravenous flat-detector computed tomography (IV FDCT) angiography in assessing hemodynamically significant cerebral vasospasm in patients with subarachnoid hemorrhage (SAH) with digital subtraction angiography (DSA) as the reference. DSA and IV FDCT were conducted concurrently in patients suspected of having symptomatic cerebral vasospasm postoperatively. The presence and severity of vasospasm were estimated according to location (proximal versus distal). Vasospasm >50% was defined as having hemodynamic significance. Vasospasms <30% were excluded from this analysis to avoid spectrum bias. Twenty-nine patients (311 vessel segments) were measured. The intra- and interobserver agreements were excellent for depicting vasospasm (*k* = 0.84 and 0.74, resp.). IV FDCT showed a sensitivity of 95.7%, specificity of 92.3%, positive predictive value of 93.6%, and negative predictive value of 94.7% for detecting vasospasm (>50%) with DSA as the reference. Bland-Altman plots revealed good agreement of assessing vasospasm between the two tests. The discrepancy of vasospasm severity was more noted in the distal location with high-severity. However, it was not statistically significant (Spearman's rank test; *r* = 0.15, *P* = 0.35). Therefore, IV FDCT could be a feasible noninvasive test to evaluate suspected significant vasospasm in SAH.

## 1. Introduction

Symptomatic cerebral vasospasm (SCV) causes delayed ischemic neurologic deficit which can be associated with poor neurologic outcomes in patients with subarachnoid hemorrhage (SAH) [1]. Cerebral vasospasm following SAH has a morbidity and mortality rate of 10% to 30% [2]. Consequently, early detection and prompt management are required before ischemic brain injury occurs by vasospasm. The current treatment regimen for symptomatic vasospasm includes primary medical and secondary endovascular treatments. With the advances in endovascular technologies, intra-arterial (IA) vasodilators infusion or transluminal balloon angioplasty (TBA) have been increasingly performed for refractory vasospasm [3, 4]. Although there is no time limit for initiation of endovascular treatment, effectiveness can be achieved with a therapeutic time window of 2 hours after developing vasospasm [5]. In particular, significant vasospasm that is refractory to medical treatment requires early endovascular intervention. However, decision-making for endovascular intervention can be time-consuming. Current imaging modalities, such as transcranial Doppler ultrasonography (TCD), magnetic resonance angiography (MRA), and computed tomography angiography (CTA), cannot be directly connected to endovascular intervention. In addition, clinical diagnostic steps to differentiate cerebral vasospasm from other medical conditions including intracranial hemorrhage, hydrocephalus, seizure, infection, or delirium can delay endovascular procedure.

Intravenous flat-detector computed tomography (IV FDCT) is an emerging technology that enables CT-like images from a C-arm biplane angiography system. Image intensifiers used to be used to produce CT-like images from C-arm system. However, concerns that arose included low dynamic range and distorted images with high contrast. The high dynamic range and higher dose efficiency of the flat panel detector can clearly delineate vasculature [6]. Psychogios et al. [7] reported that high-resolution IV FDCT images are comparable to those of multidetector row CT angiography (MDCTA). IV FDCT is feasible to estimate intra- and extracranial stenosis with cerebral angiography as the reference [8, 9]. The prominent feature of IV FDCT is its application during neurointerventional procedures. White et al. [10] and Struffert et al. [11] reported that IV FDCT is useful to detect periprocedural complications and acute cerebral infarction. In addition, subsequent endovascular procedures can be conducted after identifying unexpected complications on the same angiographic suite without patient transfer. Therefore, IV FDCT could potentially improve clinical outcome in SAH patients by providing time saving benefits in identifying and treating significant cerebral vasospasm. However, to the best of our knowledge, no statistical analysis regarding the accuracy of IV FDCT compared with cerebral angiography in estimating cerebral vasospasm has been conducted. The aim of this study was to evaluate the diagnostic accuracy of IV FDCT in measuring cerebral vasospasm >50% with digital subtraction angiography (DSA) as the reference.

## 2. Material and Methods

### 2.1. Study Design

This investigation was conducted in patients suspected of having symptomatic vasospasm after aneurysm obliteration from January 2007 to October 2012 at a single center. Thirty-four SAH patients with Fisher grade III were enrolled in this cohort. Their mean age was 59.3 ± 14.7 years and 11 (37.9%) patients were male. Hypertension and a history of smoking were found in 8 (27.6%) and 5 (17.2%) patients, respectively. The mean vasospasm occurrence was 6.8 days (range 4–12 days) after ictus. Surgical clipping of 18 aneurysms (60%) and coil embolization of 12 aneurysms (40%) were performed (Table 1). Distribution of clip counter was as 11 single clips, six double clips, and one triple clip. Five cases used mini clips and 21 cases used nominal clips. Every surgical clipping was conducted with a Yasargil-Titanium Aneurysm Clip (Aesculap AG, Tuttlingen, Germany). No larger (>20 mm) [12] or fenestration clip was used in this study.

Intravenous nimodipine (Samjin Pharm, Seoul, Republic of Korea; 20 mcg/kg/h) was administered to prevent cerebral vasospasm (Figure 2). All patients were monitored by daily TCD examinations in the neurointensive care unit. Symptomatic vasospasm was suspected in the newly developed neurological deficit (e.g., impaired or deteriorated consciousness, dysphasia, motor weakness, or sensory changes) associated with elevated TCD velocity or when the Lindegaard ratio was met [13] after excluding other causes (e.g., aneurysm rebleeding, intracranial or surgery associated hematoma, hydrocephalus, electrolyte disturbance, procedure related infarct, seizure, or infection). Confirmation and calculation of vasospasm severity were made based on DSA findings. After diagnostic angiography, IV FDCT was performed simultaneously to ascertain the change in vessel size before endovascular intervention. The images, given as maximum intensity projection (MIP) images gained by IV FDCT, were reconstructed and matched with the corresponding DSA images. If images of preoperative IV FDCT were unavailable, normal proximal arterial diameter was used as the reference to measure vasospasm severity. All vessel diameters were calculated with an electric ruler by two endovascular neurosurgeons blinded to clinical information. The following vessels were measured for analysis: both distal intracranial internal carotid artery (ICA), A1, A2, M1, M2, P1, P2, and V4, located just proximal to PICA, and the mid-point of the basilar artery. Cases of hypoplastic segments [14] or poor image quality for interpretation due to artifact were excluded for analysis. The concordance of IV FDCT with DSA was evaluated according to location (proximal versus distal). Proximal located vessels included distal ICA, M1, A1, P1, and BA. The vasospasm severity was categorized into three groups: none or mild (<30%), moderate (30–50%), and severe (>50%) [15]. Vessel narrowing >50% was defined as hemodynamically significant cerebral vasospasm [12]. Vasospastic vessels <30% were excluded from this analysis to avoid spectrum bias [8, 16]. This study was approved by the Institutional Review Board and informed consent was always obtained (number 2011-45).

### 2.2. Image Acquisition

Eighty milliliters of PAMIRAY250 contrast agent (Iopamidol; Dong kook Pharm., Seoul, Republic of Korea) with 40 mL of saline was infused through an anterior cubital vein with a flow rate of 5 mL/s. IV FDCT data acquisition was conducted according to the following protocol: 0.4 of increment, 200° of total angle, 538 of total projections, radiation dose of 35 mGy, acquisition time of 20 seconds rotation, and 18 seconds X-ray time delay. Postprocessing of the IV FDCT data was performed on a Leonardo DynaCT dedicated work station equipped with InSpace 3D software (Siemens, Erlangen, Germany) [8]. The software adapted system-specific filter algorithms to adjust beam hardening and ring artifact, radiation scatter, and truncated projections. A volume dataset of a batch of about 400 slices in a 512 × 512 matrix and voxel size of 0.1 × 0.1 × 0.1 mm^3^ was reproduced. IV FDCT raw data were reconstructed in axial, coronal, and sagittal directions of maximal intensity projection (MIP) images with various slice thicknesses, mainly 15 mm. All IV FDCT and DSA were performed with the AXIOM Artis zee Biplane system (Siemens, Erlangen, Germany).

### 2.3. Statistical Analyses

The intra- and interobserver agreements, sensitivity, specificity, positive predictive values (PPV), and negative predictive values (NPV) of IV FDCT to identify hemodynamic significant vasospasm were calculated. The Bland-Altman method was used to assess the diagnostic accuracy of IV FDCT according to location. Receiver operating characteristic (ROC) curves were estimated for defining vasospasm >50% including all vessel segments (≥30%) with DSA as the reference. Analyses were performed with SPSS version 18 (SPSS, Chicago, IL) and MedCalc software (Medcalc, Ostend, Belgium).

## 3. Results

**Figure 1:**
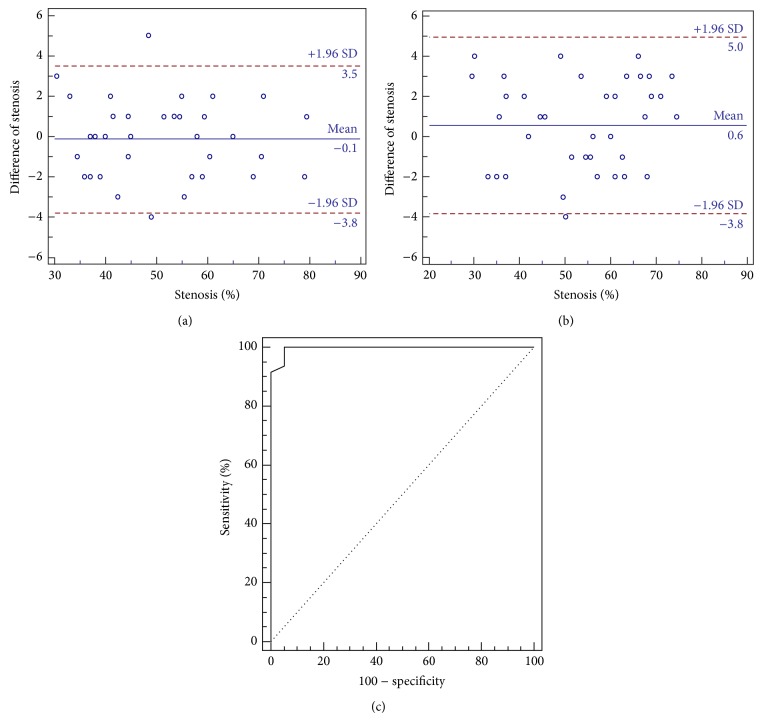
Bland-Altman plot reveals excellent concordance of measuring vasospasm severity according to locations such as proximal (a) and distal (b). (c) Receiver operating characteristic curves for detecting vasospasm (>50%) in all vessel segments (>30%). The area under the curve is 0.996. IV FDCT >48% has a sensitivity of 93.6% and specificity of 94.7% in detecting hemodynamic significant vasospasm (>50%) with DSA as the reference (proximal arteries include distal internal carotid artery, M1 segment of the middle cerebral artery, A1 segment of the anterior cerebral artery, P1 segment of the posterior cerebral artery, and basilar artery).

**Figure 2:**
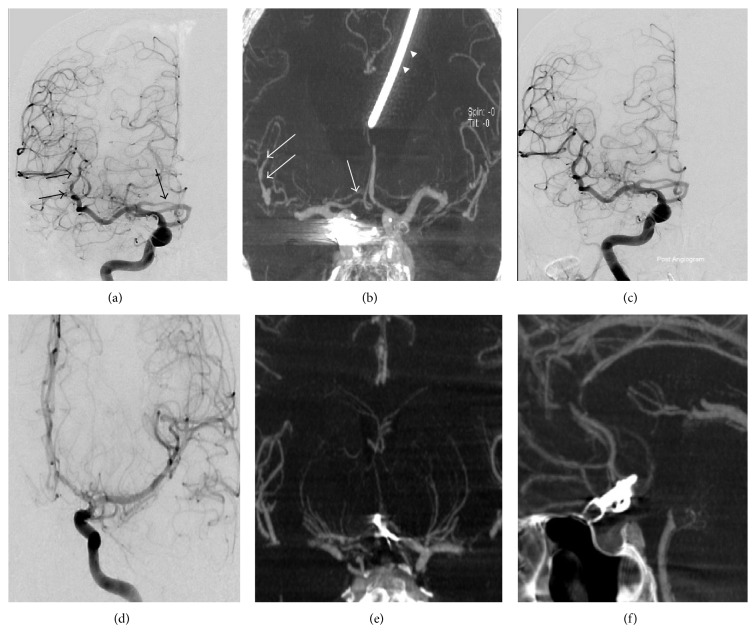
An 81-year-old female was transferred for ruptured aneurysm of the posterior communicating artery. (a and b) Digital subtraction angiography (DSA) and IV FDCT taken 5 days after the coil embolization revealed moderate vasospasm of the M2 and A1 (arrows) (arrowhead indicates a catheter of the extraventricular drainage). (c) DSA after chemical angioplasty using nimodipine shows the improvement of blood flow. A 22-year-old male was transferred for ruptured aneurysm of the anterior communicating artery. (d, e, and f) DSA and selective coronal and axial maximal intensity projection image of IV FDCT show no evidence of vasospasm.

Of 34 Fisher grade III patients suspected of having symptomatic vasospasm, 29 patients harboring 30 aneurysms demonstrated angiographic evidence of vasospasm. A total of 311 vessel segments in 29 patients were analyzed. The locations of aneurysms were the anterior communicating artery (Acom; *n* = 8, 26.7%), middle cerebral artery (MCA; *n* = 8, 26.7%), posterior communicating artery (Pcom; *n* = 5, 16.7%), basilar artery (BA; *n* = 3, 10%), and vertebral artery (VA; *n* = 1, 3.3%). Eighty-five vasospastic vessel segments (≥30% luminal narrowing) were observed (Table 2). ACA territory comprised vasospasm (*n* = 45, 53.0%) followed by MCA territory (*n* = 30, 35.3%). Hemodynamically significant vasospasm was observed in 46 vessel segments. The intra- and interobserver agreements were excellent for depicting vasospasm (*k* = 0.84 and 0.74, resp.). IV FDCT demonstrated a sensitivity of 95.7% (95% CI 85.2–99.5%), specificity of 92.3% (95% CI 79.1–98.4%), PPV of 93.6% (95% CI 82.5–98.7%), and NPV of 94.7% (95% CI 82.3–99.4%) for identifying vasospasm >50% with DSA as the reference (Table 3). Bland-Altman plots revealed good agreement of estimating vasospasm severity between IV FDCT and DSA according to location (proximal versus distal) (Figures 1(a) and 1(b)). Discrepancy of vasospasm severity (DSA-IV FDCT, %) was more notable in the distal location with higher-grade vasospasm. However, it was not statistically significant (Spearman's rank test; *r* = 0.15, *P* = 0.35). ROC curves for detecting vasospasm >50% in all vessel segments (≥30% luminal narrowing) are depicted in Figure 1(c). The area under the curve was 0.996. The cut-off value of IV FDCT >48% had a sensitivity of 93.6% (95% CI 82.5–98.7%) and specificity of 94.7% (95% CI 82.3–99.4%) in detecting vasospasm >50%. The cut-off had to be set at >46% to attain 100% sensitivity for identifying hemodynamic significant vasospasm.

## 4. Discussion and Conclusion

Early identification and treatment for symptomatic vasospasm are crucial after obliteration of cerebral aneurysms. Although medical treatment has been used as a first-line therapy [17], endovascular treatment has been performed for vasospasms that prove refractory to medical therapy. In addition, distal vasospasm can be treated by mechanical angioplasty. Santillan et al. [3] reported that balloon angioplasty decreases the need for chemical angioplasty in patients with distal cerebral vasospasm. Considering the tissue at risk concept [18], which is the penumbra area that will be restored with early reperfusion from an ischemic state, prompt endovascular intervention after diagnosis could be beneficial for significant cerebral vasospasm. Ideal radiologic test for SCV requires a shorter acquisition time, high diagnostic accuracy with fewer artifacts, subsequent connection to the endovascular procedure, and less radiation exposure. TCD, MRA, and CTA have been conducted widely for the evaluation of cerebral vasospasm. TCD has been used for screening vasospasm due to its relative simple procedure and availability [19]. Vora et al. [20] showed that only patients with low (<120 cm/s) or very high (≥200 cm/s) MCA velocities correlate with the absence or presence of the significant vasospasm. Nakae et al. [21] reported that an increase in the mean blood flow velocity ratio of the ipsilateral to contralateral MCA is more valuable than absolute flow velocity in predicting vasospasm. However, TCD has limited value in estimating vasospasm in other arteries except for MCA [22] and vasospasm under intermediate velocities (120–200 cm/s) [20]. In addition, interpretation of the result can be limited by the examiner's expertise and narrow window [23]. Accordingly, MRA or CTA has been performed to ascertain the vasospasm. Time-of-flight (TOF) MRA is the standard technique to evaluate cerebral vasculature. Grandin et al. [24] showed a good agreement between MRA and DSA in depicting vasospasm following SAH. In their study, MRA had a sensitivity of 92%, specificity of 98%, and negative predictive value of 98%. However, sensitivity for vasospasm of the ICA and MCA was 25% and 56%, respectively. Some disadvantages for the evaluation of cerebral vasospasm are metallic artifacts by clip, coil, stent, and devices of craniotomy site after operation [25] and longer acquisition time, which can be less favorable with fluctuations in neurologic conditions. In addition, overestimation of the arterial stenosis due to turbulent flow [26] or image degradation due to methemoglobin in the subarachnoid space [2] can be of concern. CTA is a fast and reliable method to detect cerebral vasospasm. Greenberg et al. [27] reported that CTA had 79.6% sensitivity, 93.1% specificity, 18.1 positive likelihood ratio, and 0.2 negative likelihood ratio in estimating cerebral vasospasm. Yoon et al. [12] showed that MDCTA had 97.5% accuracy, 98.1% sensitivity, and 98.0% specificity in detecting vasospasm over 50%. The agreement for proximal and distal segments of the vasospasm was 97.3% and 96.1%, respectively. Nevertheless, technical challenges including proper timing of bolus injection and beam-hardening effect can produce images of suboptimal quality [28]. In our study, IV FDCT showed a high sensitivity (95.7%) and specificity (92.3%) in detecting hemodynamic significant vasospasm. Discrepancies of vasospasm severity according to location, such as anterior or posterior, were not observed. Although more vasospastic vessels were underestimated in the distal location with higher grade stenosis, it was not statistically significant (*P* = 0.35). Such a phenomenon can be explained by the characteristics of the radiologic tools. Stenosis measurement by DSA may be influenced by flow velocity and contrast dilution. Accordingly, increased blood velocity due to hypertensive treatment and contrast dilution due to hypervolemic therapy may exaggerate the degree of vasospasm. In addition, IV FDCT can generate images that are more uniformly distributed than DSA [29, 30]. Therefore, IV FDCT may underestimate the degree of vasospasm of the higher stenotic vessels in the distal location compared with DSA.

Simultaneous endovascular intervention without patient transfer could be beneficial in patients with vasospasm refractory to medical treatment. Although there is no therapeutic time window for vasospasm, early intervention within 2 hours has been effective in reversing vasospasm [5]. In our institution, suspected hemodynamically significant vasospasm in SAH patients with Fisher grade III is indicated for urgent endovascular intervention. Fisher et al. [31] correlated thick blood clot of the subarachnoid space, defined as Fisher grade III, with the occurrence of the symptomatic vasospasm. Wilson et al. [32] also showed that the degree of SAH thickness was feasible to predict symptomatic vasospasm. From our experience, about 65% of SAH patients with Fisher grade III show reversal of angiographic vasospasm (≥80% of normal luminal diameter). All endovascular procedures were conducted with 2 hours after onset of symptomatic vasospasm. Accordingly, further study is necessary to see whether IV FDCT is a useful method to improve neurologic outcome in a large cohort of high-risk SAH patients for cerebral vasospasm. Another advantage of IV FDCT is that it can provide real-time scanning images. Accordingly, other medical conditions mimicking vasospasm also can be evaluated. The clinical diagnosis of the cerebral vasospasm consists of consecutive steps to exclude other conditions, such as hydrocephalus, rebleeding of the aneurysm, intracranial hematoma, hydrocephalus, cerebral infarction, electrolyte disturbance, and seizure. Thus, cerebral vasospasm can be more clearly depicted. In addition, physicians can manage adverse events during the procedure without delay. White et al. [10] reported IV FDCT as having marked value in decision making for 40.9% of the patients during periendovascular period. In their study, extensive leakage of the contrast medium through the perforation site by microcatheter, hematoma, and enlarged ventricle size was clearly evident. Adverse events during intrahospital transfer can also be prevented. Although, there was no statistical analysis about risk reduction in patient transfer from the diagnosis (CT or MRI rooms) to the treatment (angiography suite), an accident rate up to 71% can occur in intensive care unit patients during the conveyance for radiologic tests [33]. Because patient monitoring during transfer is usually accomplished by inexperienced examiners including interns, prompt, and proper management it cannot be performed in an emergent situation [34]. Therefore, IV FDCT could have the potential to decrease unnecessary complications during transfer.

IV FDCT can provide good depiction of the various vascular territories with collateral flow in a single acquisition [35, 36] and vascular calcification. Accordingly, screening with IV FDCT can reduce the procedural time and contrast dose. It can also be helpful to select treatment methods. With good spatial resolution of approximately 0.1 mm [37], IV FDCT has shown good delineation of intracranial stent [38]. Buhk et al. [39] suggested that IV FDCT can be feasible to evaluate aneurysm after stent remodeling. Kalender and Kyriakou [40] reported that IV FDCT showed better spatial resolution than MDCTA by comparing modulation transfer time (MTF) and visual estimate of bar pattern phantom according to different pixel binning. Nevertheless, increased noise level and decreased low-contrast resolution can be of concern. Regarding radiation dose, IV FDCT has a lesser or compatible radiation dose compared with that of MDCTA. Bai et al. [41] reported the lesser effective radiation dose of 1.18 mSv in IV FDCT of 20 s scan compared with 1.89 mSv in MDCTA. Considering a conventional brain CT of 60 mGy [42], IV FDCT can allow intracranial image with lesser radiation exposure of 35 mGy.

Because cerebral blood volume can be estimated with the same acquisition [11], detection of cerebral vasospasm anatomically and functionally could be obtained. Struffert et al. [43] showed a high correlation between abnormal CBV lesion on IV FDCT and stroke volume on follow-up MDCT. Nevertheless, lower temporal resolution of IV FDCT than that of conventional perfusion CT can be a limitation. Because the lower temporal resolution attributes to the broader time attencuation curve with lower peak, CBV and CBF can be overestimated [44].

Image degradation by metal artifact can be a concern in detection of cerebral vasospasm. Psychogios et al. [45] reported that long axis of a clip placed parallel or perpendicular to the rotational plane can generate metal artifact. Besides clip direction, larger clip and multiplicity are associated with image degradation [45]. The latter authors did not recommend IV FDCT as a routine radiologic test following postclipped aneurysms, because poor-quality images were observed in 30% of cases. Buhk et al. [39] also reported that IV FDCT cannot routinely replace the role of DSA in detecting residual neck after endovascular intervention. Because large coil mass causes an amorphous signal within the coil mass, accurate detection of residual filling cannot be obtained in the images of IV FDCT [39]. In our series, seven patients (24.1%) experienced poor-quality images (surgical clipping, *n* = 5; coil embolization, *n* = 2) for identifying the presence of residual neck or parent vessel patency due to metal artifacts. In particular, multiple clips were associated with image degradation. Nevertheless, the presence of vasospasm can be seen through the various reconstructed images. Motion artifact also can occur in patients of compliance. Accordingly, a newly developed algorithm for metal artifact reduction and methods to increase C-arm rotation time with decreased angular sampling are needed.

The distinctive feature of this study is that two tests were conducted simultaneously to mitigate the possibility of vessel change. Vasospasm in SAH patients tends to have a hemodynamic nature. Moreover, vessel diameters can be affected during the medical treatments by maintaining hypertensive and/or hypervolemic status. Therefore, measurement of the vessel size at a different period may produce inaccurate results.

There are some limitations in this study. First, the small number of patients and single center experience are limitations. In addition, vasospastic vessels ≥30% of the posterior circulation were not sufficiently enrolled. Second, the possibility of selection bias can be a concern. We think that clinical change in Fisher grade III SAH patients associated with elevated TCD velocity could contribute to the relative high prevalence of the cerebral vasospasm in our cohort. Nevertheless, the result might not apply to general SAH patients. Third, safety issue associated with additional contrast and radiation cannot be fully investigated. This method may result in an increased radiation dose and increased contrast rather than just specific, focused DSA runs. Accordingly, further studies are needed to elucidate the safety of the additional contrast and radiation in a large population.

In conclusion, IV FDCT could be feasible for detecting hemodynamic significant vasospasm. We do not advocate the superiority of IV FDCT more than other noninvasive tests in detecting cerebral vasospasm. We believe that IV FDCT could also be a feasible noninvasive test for the evaluation of suspected hemodynamic significant vasospasm in SAH patients who would benefit from early endovascular treatment. A multicenter study including direct comparison of IV FDCT to the other noninvasive tests is needed.

## Figures and Tables

**Table 1:** Clinical characteristics of 29 Fisher grade III SAH patients harboring 30 aneurysms.

Variables	Number (%)
Male	11 (37.9%)
Age∗, years	59.3 ± 14.7
Hypertension	8 (27.6%)
Smoking	5 (17.2%)
Location	
A-com	8 (26.7%)
Pericallosal	3 (10%)
MCABF	7 (23.3%)
M1	1 (3.3%)
P-com	5 (16.7%)
BA	3 (10%)
VA	1 (3.3%)
PICA	2 (6.7%)
Methods	
Surgical clipping	18 (60%)
Coil embolization	12 (40%)

^*^Mean ± SD.

Note: A-com indicates anterior communicating artery; BA, basilar artery; MCABF, middle cerebral artery bifurcation; M1, M1 segment of middle cerebral artery; P-com, posterior communicating artery; PICA, posterior inferior cerebellar artery; SAH, subarachnoid hemorrhage; and VA, vertebral artery.

**Table 2:** Distribution of vasospastic vessel segments (>30% luminal narrowing) demonstrated by digital subtraction angiography.

Variables	Number of vasospastic segments (%)
ICA	5 (5.9%)
A1	22 (25.9%)
A2	23 (27.0%)
M1	16 (18.8%)
M2	14 (16.5%)
P1	3 (3.5%)
P2	1 (1.2%)
BA	1 (1.2%)

Total	85

Note: ICA indicates internal carotid artery; A1, A1 segment of anterior cerebral artery; A2, A2 segment of anterior cerebral artery; M1, M1 segment of middle cerebral artery; M2, M2 segment of middle cerebral artery; P1, P1 segment of posterior cerebral artery; P2, P2 segment of posterior cerebral artery; BA, basilar artery; and V4, V4 segment of vertebral artery.

**Table 3:** Accuracy of IV FDCT in identifying hemodynamic significant vasospasm (>50%) compared with DSA.

	Seen on DSA	Not seen on DSA	Total
Seen on IV FDCT	44	3	47
Not seen on IV FDCT	2	36	38

Total	46	39	85

Note: numbers are vessel segments.
